# Frontiers in Nucleic Acid Nanotechnology

**DOI:** 10.3390/nano5020750

**Published:** 2015-05-08

**Authors:** Stephen F. Ralph

**Affiliations:** School of Chemistry, University of Wollongong, Northfields Avenue, Wollongong 2522, NSW, Australia; E-Mail: sralph@uow.edu.au; Tel.: +61-2-4221-4286; Fax: +61-2-4221-4287

This Special Issue of *Nanomaterials* highlights innovative work from around the world focused on harnessing the physical, chemical and topological properties of nucleic acids. It contains a mixture of articles examining composite materials that combine nucleic acids with carbon nanotubes (CNTs) or metallic nanoparticles, as well as theoretical investigations, and studies directed towards applications in a variety of areas. The latter include nanophotonics, new methods of DNA sequencing, and the development of new antibacterial agents or drug delivery vehicles. Highlights of the publications appearing in this Special Issue are discussed in the following paragraphs.

Kazuo Umemura [1] has provided a timely and comprehensive review article focused on nanobiotechnological applications of materials produced by hybridization of nucleic acids with CNTs. Since such materials were first reported a little over 10 years ago, there have been over 200 studies published in this area. The prevalence of investigations directed towards finding methods for enhancing the solubility of CNTs, or enabling their separation and purification is highlighted, before a selection of articles are described which exemplify some of the work currently being undertaken to develop new applications for hybrid DNA/CNT materials. Amongst these studies the preparation of new materials for sensing of a range of analytes, such as other DNA molecules, neurotransmitters, glucose, hormones, various gases and even bacterial cells, is a common theme. Other studies referenced in the review describe the potential of DNA/CNT hybrids for destroying cancer cells via thermal ablation, and highlight that the preparation of hybrid materials consisting of DNA and graphene is a rapidly emerging area.

The development of a new method for preparing hydrogels composed of multi-walled carbon nanotubes (MWNTs) and two types of DNA is the topic of the study undertaken by Zinchenko and co-workers [2]. A feature of this investigation was the use of relatively low molecular weight DNA extracted from salmon milt to initially produce dispersions of MWNTs. Since salmon milt is readily available in significant quantities as a by-product of the fishing industry, it offers environmental advantages for applications requiring large quantities of soluble nanotubes. Hydrogels were produced from the initial DNA/MWNT dispersions by taking advantage of the fragments of adsorbed DNA still exposed to the surrounding solution. These fragments were chemically crosslinked in a process using DNA molecules with a much higher molecular weight, and ethylene glycol diglycidyl ether. Using this procedure homogeneous hydrogels were obtained that exhibited similar swelling properties to that of DNA hydrogels containing no MWNTs. The hybrid DNA/MWNT hydrogels were shown to be significantly stronger than those not containing nanotubes in experiments measuring their extent of shrinkage in the presence of high concentrations of NaCl.

DNA obtained from salmon milt was also used in research led by Tomomi Takeshima [3]. In this case single stranded DNA containing approximately 60 nucleotides was used as the stabilizing agent in a procedure for preparing silver nanoparticles ranging in size between 2 and 20 nm. Zeta potential measurements confirmed that the surfaces of the nanoparticles were covered in negatively charged DNA molecules. The nanoparticles were shown to inhibit the growth of both a Gram-positive and a Gram-negative bacterium in a concentration dependent manner, most likely owing to the release of free Ag(I) ions. Of the two types of bacteria examined, the nanoparticles proved to be more effective towards the Gram-negative *E. coli*. As a demonstration of the potential application of their discovery, the researchers immobilized the Ag/DNA nanoparticles onto the surfaces of cotton fabrics, which had been previously modified using a cationic polymer in order to facilitate electrostatic binding of the nanoparticles. The resulting materials were shown to exhibit significant bacteriostatic and bactericidal properties in standardized tests.

One of the most exciting areas of nanotechnology research centers on the development of carrier systems for facilitating the delivery of therapeutic agents selectively to specific cells or molecular targets. As part of their efforts in this area, Kocabey and co-workers [4] have described the preparation of modified DNA nanotubes containing siRNA, folic acid or fluorescent dyes, as well as the results of experiments designed to explore the stability of these systems, and their level of uptake by HeLa cells. Initial experiments focused on DNA nanotubes measuring approximately 27 nm long, and with a diameter of ~6 nm, that were prepared using single-stranded DNA consisting of 42 nucleotides. Unfortunately, these nanotubes were incapable of entering the cytoplasm of HeLa cells and delivering their payload in order to effect gene silencing and altered gene expression. This prompted a series of insightful investigations into factors that can dramatically affect the stability of DNA-based drug delivery systems. It was shown that increasing the size of the DNA nanotubes, by doubling the number of nucleotides present in the initial DNA tiles, significantly increases their lifetime, even when in contact with buffers containing relatively low concentrations of stabilizing divalent cations. The authors conclude their article by providing a warning concerning interpretation of the results of drug-delivery experiments when the stability of the transport vehicle has not been fully investigated.

Cervantes-Salguero and co-workers [5] report the preparation of flexible DNA building blocks using the DNA origami method, as well as their self-assembly into clusters of different sizes and shapes, including triangles, squares and pentagrams, on the surfaces of pieces of mica. In a series of elegant experiments followed using atomic force microscopy (AFM), the researchers show how addition of sodium ions facilitates the disassembly of the clusters, and their subsequent reconfiguration into different shapes via diffusion of the individual blocks on the surface of the mica. AFM was also used to examine the effect of varying the length of flexible regions within the DNA building blocks on the final distribution of clusters, thereby providing insights into how this approach could be modified to yield clusters of other sizes and shapes in future.

Titration of solutions containing non-covalent DNA/MWNT complexes, with didodecyl-dimethylammonium bromide (DDAB), resulted initially in the formation of sticky precipitates, which re-dissolved when excess cationic surfactant was added. Tardani and La Mesa [6] have provided a comprehensive report on their investigations of these mixtures, which were conducted using a range of techniques. These included optical spectroscopy and microscopy, dynamic light scattering, and both ionic conductivity and zeta potential measurements. From the results obtained a (pseudo)-binary phase diagram was obtained, and the properties of the systems rationalized by considering a model in which the DNA/MWNT complexes were considered to be rigid, rod-like polyelectrolytes. Significant variations in behavior were observed compared to what is seen with the analogous surfactant dodecyltrimethylammonium bromide (DTAB), which only contains one hydrophobic chain. For example, precipitate re-dissolution is far less extensive when DTAB is added, consistent with it interacting less effectively with DNA/MWNT complexes. These insights have important implications for researchers interested in understanding how other biomolecules might bind to DNA complexes.

The ability to use DNA as a template for the controlled assembly of clusters of sliver atoms is the topic of a detailed review by Gwinn and co-workers [7]. Drawing on their own work and that of other researchers, the authors discuss methods for isolation and purification of DNA/Ag composites, as well as a number of methods, including electrospray ionization mass spectrometry and inductively coupled plasma atomic emission spectrometry, for analyzing the composition of these materials. It is revealed that DNA/Ag materials can be produced using a variety of nucleic acid structures, including the ubiquitous Watson-Crick double helical form, and different hairpin structures. The authors also explain that the observation of a single absorption band in the visible or near IR region supports the conclusion that the clusters have a rod shape, as opposed to globular. Furthermore the reason why certain fluorescence colors are more readily observed in DNA/Ag materials is accounted for by proposing that clusters containing specific (“magic”) numbers of silver atoms exhibit enhanced stability compared to those with fewer or greater numbers of atoms. A brief description of the chiral properties of DNA/Ag materials is provided, along with a discussion of as yet unanswered questions concerning their preparation and properties.

Li and co-workers [8] review the state of the art with respect to our understanding of the effects of external forces on DNA structure. Advances in this area have been made possible through the advent of a number of new nano-manipulation and single-molecule techniques. The latter, in particular, have enabled researchers to explore how single stranded and double stranded DNA molecules with different base sequences respond to applied mechanical forces. Significant progress has now also been made in an effort to understand the effects of numerous factors, such as the presence of simple cations, on the strength of DNA-DNA interactions. The importance of solvent molecules in mediating such interactions is also addressed, and a limited discussion of investigations performed using DNA structures other than B-form DNA also presented. Developing a more sophisticated understanding of how DNA responds to external forces is critical if we wish to take advantage of its unique structural and chemical properties in nanotechnology, and in order to fully comprehend how DNA interacts with other complex multi-protein machines as it performs its biological functions inside cells.

The search for more rapid and reliable methods of DNA sequencing is an important objective as researchers seek to develop new biotechnological devices and products. One method of DNA sequencing that has attracted considerable interest in recent years involves the use of nanopores composed of the protein alpha-hemolysin. Manara and co-workers [9] describe the results of molecular dynamics simulations to examine how effectively a nucleotide would be captured if it were generated at different distances from the mouth of the nanopore, through the action of an exonuclease on an oligonucleotide. The author’s simulations demonstrate that capture is most effective when the nucleotide is released close to the nanopore, and that the initial orientation of the nucleotide and its position relative to the center of the mouth of the protein are less important factors. Several amino acids that play a key role in preventing access of the nucleotide to the nanopore are also highlighted.

The above articles showcase the breadth of nanotechnological research currently being undertaken throughout the world that seeks to take advantage of the many unique features of DNA. We hope that you enjoy reading this Special Issue, and look forward to your contributions to this journal in this fascinating area in the years ahead.
